# Comprehensive Stiffness Modeling and Evaluation of an Orthopedic Surgical Robot for Enhanced Cutting Operation Performance

**DOI:** 10.3390/biomimetics10060383

**Published:** 2025-06-08

**Authors:** Heqiang Tian, Mengke Zhang, Jiezhong Tan, Zhuo Chen, Guangqing Chen

**Affiliations:** College of Mechanical and Electronic Engineering, Shandong University of Science and Technology, Qingdao 266590, China; tianhq26@sdust.edu.cn (H.T.); 17853532019@163.com (M.Z.); tanjiezhong2023@163.com (J.T.); 18865701866@163.com (Z.C.)

**Keywords:** orthopedic surgical robot, stiffness modeling, virtual joint method, joint stiffness identification, stiffness distribution

## Abstract

This study presents an integrated stiffness modeling and evaluation framework for an orthopedic surgical robot, aiming to enhance cutting accuracy and operational stability. A comprehensive stiffness model is developed, incorporating the stiffness of the end-effector, cutting tool, and force sensor. End-effector stiffness is computed using the virtual joint method based on the Jacobian matrix, enabling accurate analysis of stiffness distribution within the robot’s workspace. Joint stiffness is experimentally identified through laser tracker-based displacement measurements under controlled loads and calculated using a least-squares method. The results show displacement errors below 0.3 mm and joint stiffness estimation errors under 1.5%, with values more consistent and stable than those reported for typical surgical robots. Simulation studies reveal spatial variations in operational stiffness, identifying zones of low stiffness and excessive stiffness. Compared to prior studies where stiffness varied over 50%, the proposed model exhibits superior uniformity. Experimental validation confirms model fidelity, with prediction errors generally below 5%. Cutting experiments on porcine femurs demonstrate real-world applicability, achieving average stiffness prediction errors below 3%, and under 1% in key directions. The model supports stiffness-aware trajectory planning and control, reducing cutting deviation by up to 10% and improving workspace stiffness stability by 30%. This research offers a validated, high-accuracy approach to stiffness modeling for surgical robots, bridging the gap between simulation and clinical application, and providing a foundation for safer, more precise robotic orthopedic procedures.

## 1. Introduction

In recent years, the integration of robotic systems into orthopedic surgery has advanced rapidly, particularly with the developments over the past decade [1,2]. Modern surgical robots increasingly feature high-precision cutting tools capable of performing complex osteotomies, such as spinal fusion and joint replacements. These systems are gradually assuming greater responsibility for bone resections traditionally performed by surgeons. However, the high cutting forces involved can lead to structural deformations in the robot, primarily due to the anisotropic characteristics of bone and the risk of thermal damage. These multi-axis dynamic forces challenge the balance between the flexibility needed for surgical adaptability and the stiffness required for precision. Studies have shown that robotic assistance can improve cutting accuracy by 20–30% and reduce operation time by 5–10% [3,4]. Thus, robotic systems must offer both high flexibility and sufficient stiffness within the surgical workspace to ensure optimal performance.

Stiffness requirements vary significantly across surgical scenarios. For example, in spinal surgeries such as pedicle screw fixation, optimal stiffness ranges from 300 to 500 N/mm to balance structural stability and neural safety—excessive stiffness may cause screw loosening or fractures, while insufficient stiffness may lead to micromotion exceeding 0.5 mm and increase the risk of nerve injury [5]. In minimally invasive decompression, robotic arms must alternate between “rigid-flexible” states: high stiffness (≥800 N/mm) ensures precise bone grinding, while low stiffness (≤200 N/mm) supports safe navigation near nerve tissue [6]. In joint replacement surgeries, femoral osteotomy requires stiffness above 1200 N/mm to prevent angular deviation, and prosthesis alignment typically demands 1000–1500 N/mm to endure multi-cycle gait loads with a variation coefficient under 10% to avoid stress shielding and bone loss [7]. These examples underscore the importance of task-specific stiffness modulation in surgical robot design.

Stiffness critically influences robotic machining performance by affecting cutting precision, force control, speed, and overall surgical safety [8]. Clinical reports indicate that 23% of revision surgeries in robotic knee replacements are due to inadequate stiffness management [9]. High stiffness minimizes vibration and deformation, ensuring more accurate cuts and stable force application, thereby reducing unintended tissue damage. Consequently, thorough stiffness analysis is essential to meet surgical safety standards, such as FDA/IEC requirements that mandate stiffness errors remain within ±15% [10].

Despite widespread adoption, many robotic platforms lack detailed stiffness performance data. For instance, the MAKO robot (Stryker) shows an average end-effector stiffness of ~85 N/mm under static conditions [11], while the Rosa Spine system (Zimmer Biomet) achieves ~120 N/mm during screw insertion [12]. In contrast, our proposed method enables sub-millimeter stiffness prediction across varying postures, potentially improving stiffness performance by 30% and response speed by 40%, highlighting its clinical value.

End-effector stiffness varies spatially within the surgical workspace, leading to non-uniform distributions that require the mapping of iso-stiffness regions and the identification of stable operational zones. This is further complicated by dynamic interactions between the cutting tool and bone. For instance, cutting-induced thermal effects and soft tissue viscoelasticity can significantly reduce local stiffness [13]. Bone exposed to temperatures above 45 °C may soften irreversibly, losing 15–20% of its effective stiffness. Thermo-mechanical simulations (e.g., ANSYS) show nonlinear stiffness attenuation with temperature gradients. Simultaneously, viscoelastic tissues (e.g., cartilage) require adaptive impedance control to maintain appropriate virtual stiffness and damping responses [14].

Recent research has increasingly emphasized stiffness modeling and joint stiffness identification to address these challenges quantitatively [15]. Current modeling methods fall into three categories: finite element analysis (FEA), matrix structural analysis (MSA), and the virtual joint method (VJM). FEA offers high accuracy and is primarily used during design stages, though it is computationally intensive for complex 3D models [16,17]. Its validity has been confirmed experimentally by researchers such as Corradini et al. [18] and Trochimczuk et al. [19]. MSA integrates FEA principles and strain energy theory but sacrifices accuracy and is less suitable for deriving Cartesian stiffness matrices or analyzing irregular structures [20,21,22]. These limitations highlight the importance of considering tool–tissue interactions, which directly influence intraoperative force control.

Screw theory-based methods calculate stiffness matrices from strain energy and elastic deformation, as demonstrated by Yang et al. [23] and further refined by Li et al. [24] and Cao et al. [25], achieving strong correlation with FEA results. VJM, in contrast, introduces virtual elastic joints into a rigid model and uses the Jacobian matrix to derive stiffness. While less precise than FEA, VJM is computationally efficient and widely adopted for stiffness modeling [26,27]. Its application has been expanded to serial and parallel robots by Salisbury [28], Gosselin [29], and Hoevenaars [30], incorporating flexible, driven, and zero-stiffness joints. Recent advances by Huang et al. [31], Sheng et al. [32], and Anatol et al. [33] have integrated external load effects and developed nonlinear stiffness models for systems with passive joints.

Accurate stiffness modeling also depends on high-precision measurement tools, including laser trackers, optical coordinate measuring machines (CMMs), and laser interferometers. These systems enable real-time monitoring of displacement, trajectory, and angular variation, essential for stiffness identification [34,35]. Prior studies by Dumas et al. [36], Kamali et al. [37], Nubiola et al. [38], Matteo et al. [39], Hovland et al. [40], Zanchettin et al. [41], and Kamali et al. [42] have demonstrated the effectiveness of these tools in joint stiffness measurement.

In summary, while VJM has emerged as the most efficient stiffness modeling method, dynamic joint stiffness testing remains essential for accurate parameter identification. This study provides a comprehensive analysis of stiffness distribution in orthopedic surgical robots and demonstrates up to 30% improvement in stiffness stability. A unified stiffness model incorporating end-effector, cutting tool, and force sensor stiffness is developed. The virtual joint method, combined with joint stiffness identification via laser trackers, is employed for end stiffness analysis. The simulation and experimental results illustrate the spatial distribution of stiffness during cutting, offering valuable insights for improving surgical stability and performance.

This paper is structured as follows: Section 2 presents the stiffness model of the cutting system; Section 3 details joint stiffness identification; Section 4 discusses simulation analysis; Section 5 validates the integrated model through surgical cutting experiments; and Section 6 describes the experimental stiffness measurement process. These findings provide both theoretical support and a clear clinical translation path, aligning with Chinese medical device development protocols.

## 2. Stiffness Modeling of Cutting Systems for Orthopedic Surgical Robots

**Figure 1:**
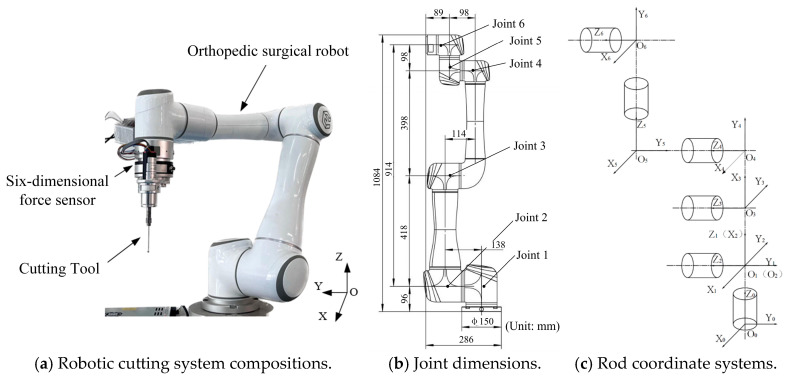
Joint dimensions and connecting rod coordinate systems of robotic cutting system for orthopedic surgery.

The cutting system consists of the robot body, a six-axis force sensor, and the cutting tool, as illustrated in Figure 1a–c, which depict the robot joint coordinate system positions, joint angle conventions, and detailed joint dimensions. The robot utilizes precise motion control and positioning technologies to guarantee accurate manipulation and control of the cutting tool throughout the bone cutting procedure.

The stiffness of the orthopedic surgical robot’s cutting system significantly influences critical aspects such as cutting accuracy, force control, cutting speed, and overall cutting quality and safety. On the premise that the connecting rod is not deformable, this model primarily encompasses the robot end stiffness, cutting tool stiffness, and force sensor stiffness. The overall stiffness of the orthopedic surgical robot cutting system can be represented by the following equation:

(1) $$K^{-1}=K_{m}^{-1}+K_{j}^{-1}+K_{s}^{-1}$$

where K denotes the robot cutting system stiffness. $K_{m}$ denotes the robot end stiffness. $K_{j}$ denotes the cutting tool stiffness. $K_{s}$ denotes the force sensor stiffness.

According to the traditional stiffness model proposed by Salisbury, the robot end stiffness depends on the stiffness of the robot joints and the Jacobi matrix of the position. Joint stiffness is an intrinsic characteristic of the robot, and it is determined after the robot is manufactured. To obtain a realistic robot stiffness matrix, the stiffness of each robot joint must be experimentally identified.

### 2.1. Stiffness of Cutting Tool and Sensor

**Figure 2:**
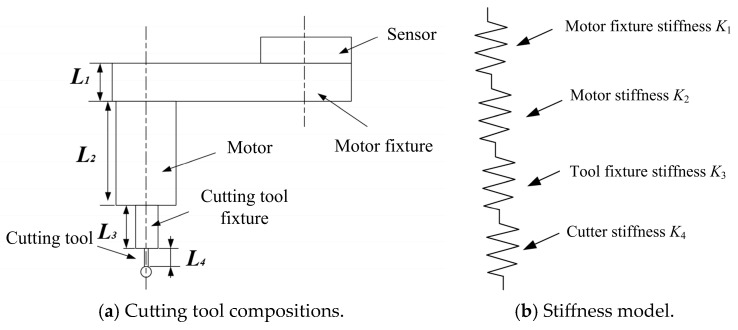
Cutting tool compositions and their corresponding stiffness model.

The cutting tool consists of a clamping part and a working part, as illustrated in Figure 2. The force sensor stiffness value is denoted as indicated and can be obtained from the manufacturer’s manual. During bone cutting, the motor, tool fixture, and cutting tool undergo deformation due to the reaction force from the bone. Considering the cutting tool body as a serial structure, it can be represented as a sum of cylindrical bending stiffness of different diameters. The expression for cylindrical bending stiffness is as follows:

(2) $$K_{i}=3E_{i}I_{i}{\left(L_{i}^{3}\right)}^{-1}$$

where $K_{i}$ denotes the equivalent stiffness of the cutting tool in N/mm. $E_{i}$ denotes the cutting modulus of each cylindrical part in MPa. $I_{i}$ denotes the polar moment of inertia of each cylinder of different diameters on the cutting tool in m^4^. $L_{i}$ denotes the effective length in mm.

The overall cutting tool stiffness can then be expressed as a summation of multiple cylindrical bending stiffness, as shown below:

(3) $$K_{t}^{-1}=\sum_{i=1}^{n}{K_{i}}^{-1}=\sum_{i=1}^{n}L_{i}^{3}{\left(3E_{i}I_{i}\right)}^{-1}$$

where *i* = 1, 2, …, *n*. *n* is the number of cylinders.

### 2.2. Robot End Stiffness

The robot end stiffness refers to the ability of the orthopedic surgical robot’s end-effector to resist deformation under load. When a load *F* is applied to the robot, the resulting deformation variable is denoted as *X*. For small deformations (*X* is small), the robot’s stiffness is linearly related to the added load through the relationship:

(4) $$F=K_{m}X$$

This relationship can be expressed in matrix form as follows:

(5) $$\left[\begin{array}{c} F_{x}\\ F_{y}\\ F_{z}\\ M_{x}\\ M_{y}\\ M_{z} \end{array}\right]=\left[\begin{array}{cccccc} K_{11} & K_{12} & K_{13} & K_{14} & K_{15} & K_{16}\\ K_{21} & K_{22} & K_{23} & K_{24} & K_{25} & K_{26}\\ K_{31} & K_{32} & K_{33} & K_{34} & K_{35} & K_{36}\\ K_{41} & K_{42} & K_{43} & K_{44} & K_{45} & K_{46}\\ K_{51} & K_{52} & K_{53} & K_{54} & K_{55} & K_{56}\\ K_{61} & K_{62} & K_{63} & K_{64} & K_{65} & K_{66} \end{array}\right]\left[\begin{array}{c} d_{x}\\ d_{y}\\ d_{z}\\ \delta_{x}\\ \delta_{y}\\ \delta_{z} \end{array}\right]$$

where *F* is a 6-vector matrix containing load and torque in three directions of the robot. *X* is a 6-vector matrix containing position change and torsion change in three directions of the robot. $K_{m}$ is called the robot end stiffness matrix.

Assuming that the robot is subjected to a joint moment τ, the elastic deformations produced by each joint of the robot are represented by $d_{q}$. The joint stiffness of each joint of the robot is denoted as $K_{q}$. This relationship can be written as follows:

(6) $$\tau =K_{q}d_{q}$$

where $\tau =diag\left(\tau_{1},\tau_{2},\tau_{3},\tau_{4},\tau_{5},\tau_{6}\right)$.

$d_{q}={\left[\begin{array}{cccccc} d_{q1} & d_{q2} & d_{q3} & d_{q4} & d_{q5} & d_{q6} \end{array}\right]}^{T}$.

$K_{q}=diag\left(K_{q1},K_{q2},K_{q3},K_{q4},K_{q5},K_{q6}\right)$.

Considering the robot kinematics, the relationship between the robot end position change and the robot joint angle of rotation can be derived:

(7) $$X=J(q)d_{q}$$

The relationship between the robot joint moment and the robot end force is given by the following:

(8) $$\tau =J^{T}(q)F$$

By combining Equations (4) and (6)–(8), the following equation is obtained:

(9) $$K_{m}={\left(J^{T}(q)\right)}^{-1}K_{q}J^{-1}(q)$$

The motion Jacobi matrix of the robot is represented by J(q), and the force Jacobi matrix is denoted as $J^{T}(q)$. Let $C_{m}$ be the robot end flexibility matrix, and the relationship between the robot flexibility matrix and the robot operation stiffness matrix is expressed as follows:

(10) $$C_{m}=K_{m}^{-1}$$

Thus, the robot end flexibility matrix can be used instead of the robot end stiffness matrix, leading to the following relationship:

(11) $$C_{m}=J(q)K_{q}^{-1}J^{T}(q)$$

Using the flexibility matrix instead of the stiffness matrix avoids Jacobi matrix inversion, resulting in reduced computational effort.

### 2.3. Robot Joint Stiffness

The joint stiffness matrix *K_q_* of the robot can be obtained by incorporating the stiffness of each joint into the following equation:

(12) $$K_{q}=\left[\begin{array}{cccccc} K_{q1} & & & & & \\ & K_{q2} & & & & \\ & & K_{q3} & & & \\ & & & K_{q4} & & \\ & & & & K_{q5} & \\ & & & & & K_{q6} \end{array}\right]$$

Using Equations (6)–(8) and (12), the following equation can be obtained:

(13) $$X=J(q)K_{q}^{-1}J^{T}(q)F$$

Let the flexibility of each joint of the robot be *C_q_*_1_, *C_q_*_2_, *C_q_*_3_, *C_q_*_4_, *C_q_*_5_, and *C_q_*_6_, then the joint flexibility of the robot is represented as follows:

(14) $$C_{q}=\left[\begin{array}{cccccc} C_{q1} & & & & & \\ & C_{q2} & & & & \\ & & C_{q3} & & & \\ & & & C_{q4} & & \\ & & & & C_{q5} & \\ & & & & & C_{q6} \end{array}\right]$$

The robot flexibility is obtained by taking the reciprocal of the robot stiffness, and the relationship between robot flexibility and stiffness is given by the following:

(15) $$C_{q}=K_{q}^{-1}$$

By substituting Equation (15) into Equation (13), the relationship between the deformation *X* and the flexibility matrix can be obtained as follows:

(16) $$X=J(q)C_{q}J^{T}(q)F$$

## 3. Joint Stiffness Identification of Orthopedic Surgical Robot

### 3.1. Robot Joint Stiffness Identification Experiment

**Figure 3:**
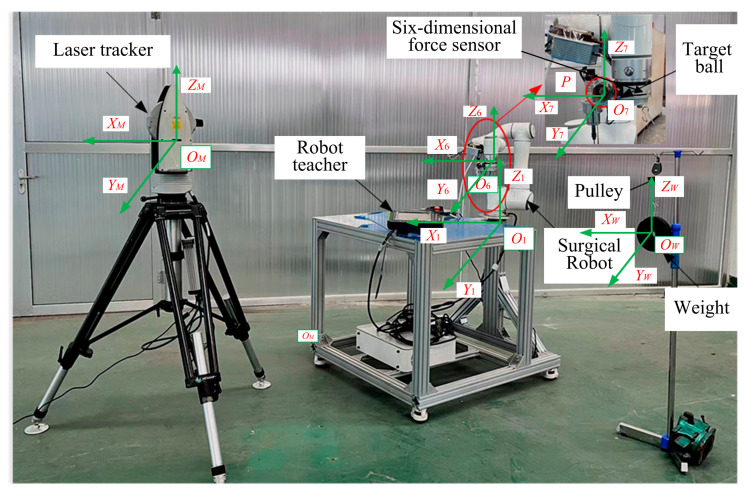
Joint stiffness identification experiment setup of robotic cutting system for orthopedic surgery.

Equation (16) clearly demonstrates that suspending a heavy object induces deformation in the robot structure. To investigate and differentiate the joint stiffness characteristics of the robot, precision instrumentation is required to measure the resulting displacement changes under load. The experimental setup designed for this purpose is illustrated in Figure 3.

This setup includes a six-axis collaborative robot with a maximum payload capacity of 6 kg, a Leica AT960 laser tracker (angular accuracy: ±15 μm + 6 μm/m; distance measurement accuracy: ±0.5 μm/m with AIFM), and a six-axis force/torque sensor (accuracy: 0.1% FS; measurement range: 200 N and 8 Nm along all three axes). The loading system comprises standardized weights (1 kg to 3.5 kg, divided into six groups), pulleys, and suspension wires. A reflective target ball is rigidly affixed to the robot’s end-effector, enabling the laser tracker to capture its 3D spatial coordinates at a sampling rate of 1000 Hz.

The experimental procedure involves positioning the robot in predefined postures, then suspending calibrated weights from the end-effector using thin wires and pulleys to apply directional forces along the x-, y-, and z-axes. The laser tracker measures the resulting end-effector displacements, while the six-axis force/torque sensor concurrently records the applied loads. This synchronized acquisition of force and displacement data facilitates accurate identification of the robot’s joint stiffness under controlled loading conditions. For simplification, the effects of torque and angular rotations are neglected in this study.

To ensure precise tracking of the robot’s posture during loading, joint angles are continuously monitored using high-resolution encoders (resolution: ±0.01°) embedded within each joint. These joint angle values are retrieved in real time via the robot controller interface and logged at each measurement point for theoretical stiffness modeling.

The displacement variations in the end-effector (Δx, Δy, Δz) are synchronously acquired using the Leica AT960 system. Its displacement resolution of ±0.5 μm/m ensures reliable capture of microscale deformations, which is critical for accurate stiffness identification in surgical robotic applications.

The force data from the six-axis sensor are time-aligned with the displacement measurements to compute stiffness values along the Cartesian axes at each robot configuration. By incorporating joint angle feedback and utilizing the robot’s Jacobian matrix, the end-effector stiffness is mapped into joint space, thereby enabling validation of the theoretical stiffness model and identification of joint-level compliance.

Since the displacement data collected by the laser tracker are referenced to its local coordinate frame, coordinate transformation is required to express these measurements relative to the robot’s base frame. Furthermore, due to potential offsets between the center of mass of the tool, target ball, and suspended weights, an additional transformation is applied to unify both the displacement and force vectors in the end-effector coordinate system. The transformation process includes the following steps:

(1) Conversion between the laser tracker and the robot base coordinate system

The center position of the target ball in the robot base coordinate system *O*_1_ can be represented as $P_{1}^{i}\left(i=1,2,3,\cdots ,N\right)$, where N denotes different positions in the robot base coordinate system *O*_1_. Similarly, the center position of the target ball in the laser tracker coordinate system *M* can be expressed as $P_{M}^{i}\left(i=1,2,3,\cdots ,N\right)$. By moving the robot to two different positions *m* and *n*, the deviation under the robot base coordinate system *O*_1_ is calculated as follows:

(17) $$\Delta P_{1}^{mn}=\Delta P_{1}^{m}-\Delta P_{1}^{n}$$

Similarly, the deviation of the center of the target ball P in laser tracker coordinates *M* can be measured as follows:

(18) $$\Delta P_{M}^{mn}=\Delta P_{M}^{m}-\Delta P_{M}^{n}$$

Since the two target balls in different positions share the same distances in both the robot base coordinate system *O*_1_ and the laser tracker coordinate system *M*, the following relationship holds:

(19) $${\left\|\Delta P_{1}^{mn}\right\|}_{2}={\left\|\Delta P_{M}^{mn}\right\|}_{2}$$

where $\Delta P_{M}^{m}$ and $\Delta P_{M}^{n}$ are the results of laser tracker measurements. By using Equation (19), the coordinates of the target ball center *P* in the robot end coordinate system *O*_6_ can be obtained by the least-squares method.

(2) Actual deformation *X* in the robot end coordinate system *O*_6_

The actual deformation *X* of the robot in the robot end coordinate system *O*_6_ can be obtained by considering a new coordinate system *O*_7_ established at the center of the target ball (Figure 3). The transformation matrix relationship between coordinate systems *O*_7_ and *O*_6_ can be expressed as follows:

(20) T76=R76P7601

Since the displacement change caused by the weight is small, it can be treated as a differential change, leading to the following:

(21) X=R7T6−R7T6⋅SP76O3×3R7T6−1dX7

(3) Load *F* in the robot end coordinate system *O*_6_

To deform the robot, a weight is added to its end, and a new coordinate system *O_w_* is established at the location where the weight is added (Figure 3). The transformation relations Rw6 and Pw6 from the coordinate systems *O*_6_ to *O_w_* can be obtained by measurement. Considering the direction of gravity is vertically downward, gravity can be described in the robot base coordinate system *O_w_* by the following expression:

(22) $$F_{w}={\left[\begin{array}{cccccc} F_{x} & F_{y} & F_{z} & 0 & 0 & 0 \end{array}\right]}^{T}$$

Then, the force F in the robot end coordinate system *O*_6_ can be calculated as follows:

(23) F=RwT6O3×3−RwT6⋅SPRwT6−1Fw

(4) Solving the robot joint flexibility matrix

As the laser tracker can only measure displacements in the *x*-, *y*-, and *z*-directions, and the robot end is less affected by moments and difficult to measure, the impact of moments can be disregarded. Therefore, based on Equation (16), the relationship between the applied force and the amount of displacement change can be rewritten as follows:

(24) $$\left[\begin{array}{l} dx\\ dy\\ dz \end{array}\right]=\left[\begin{array}{cc} I_{3\times 3} & O_{3\times 3} \end{array}\right]J(q)\cdot C_{q}\cdot J^{T}(q)\cdot F$$

In the stiffness discrimination experiments, the robot pose is chosen to avoid both the robot being in a singular position and the central axes of the robot’s joints being parallel to each other, which makes it difficult to distinguish which joint is responsible for a given deformation, thus reducing the accuracy of the joint stiffness obtained from the discrimination. The inverse of the Jacobi matrix condition number can be used as a measure of the distance from the singularity, which is known as the dexterity $K_{c}$ of the robot. The formula is given below:

(25) $$K_{c}=\frac{1}{K_{F}(J)}$$

(26) $$K_{F}(J)=\frac{1}{n}\sqrt{tr(J_{N}\cdot J_{N}^{T})}\cdot \sqrt{tr{\left(J_{N}\cdot J_{N}^{T}\right)}^{-1}}$$

In Equation (26), $K_{F}(J)$ is the Jacobi matrix condition number obtained based on the Frobenius paradigm of the Jacobi matrix; n is the dimension of the Jacobi matrix; $tr(J_{N})$ is the trace of the Jacobi matrix, and $J_{N}$ is the canonical standard type of the Jacobi matrix, and the introduction of the canonical standard type can solve the problem of the non-uniformity of the units inside the Jacobi matrix.

From Equation (25), it can be seen that 0 < $K_{c}$ ≤ 1. When $K_{c}$ = 1, all the singular values of the Jacobi matrix are equal; at this time, the end tool is far away from the singularity, and the operation flexibility and control accuracy are the best. When $K_{c}$→0, the minimum singular value of the Jacobi matrix tends to zero or the maximum singular value tends to infinity; at this time, the end tool is close to the singularity, and the flexibility and the control accuracy are significantly reduced. Therefore, the actual robot poses selected for the stiffness discrimination experiments in this paper avoid the singular poses, and these positions can cover a representative range of work throughout the surgical procedure. The joint angles and dexterity of five different poses in the surgical workspace are recorded in Table 1.

The stiffness identification experiment selected five postures primarily to balance experimental feasibility and representativeness. These five postures correspond to the most typical and mechanically significant joint positions encountered during surgery, effectively capturing key variations in stiffness characteristics. Considering constraints such as experimental duration and resource availability, increasing the number of samples would significantly raise complexity and data processing difficulty, potentially affecting the experiment’s controllability and repeatability. Although the sample size is relatively limited, preliminary statistical analysis and simulation validation confirmed that these five postures sufficiently reflect the main trends of stiffness variation in surgical scenarios, ensuring the representativeness and validity of the results.

### 3.2. Joint Stiffness Identification Results

The robot joint stiffness identification process consists of several key steps. First, the initial position *X*_1_ of the target marker is recorded. A known weight is then applied to the robot’s end-effector, and the resulting position *X*_2_ of the marker is measured. The displacement change under the coordinate system of the target ball *O*_7_ is defined as Δ*X*, which is subsequently transformed into the robot’s end-effector coordinate system *O*_6_ to obtain the actual end displacement *X* under loading. This procedure is repeated with varying weights at the end-effector.

To account for pose-dependent stiffness characteristics, the identification process is conducted across multiple robot configurations, as detailed in Table 1. The experimental results—measured displacement changes under six different loads across five robot postures—are summarized in Table 2. Analysis of these results reveals that displacement changes in the x- and y-directions are minimal. This is primarily due to the applied load being oriented vertically downward, leading to the most significant deformation occurring in the z-direction.

Based on the robot stiffness identification experiments, displacement changes under different robot configurations were obtained, enabling the identification of joint stiffness parameters using the least squares method, as summarized in Table 3. When both the robot’s posture and joint stiffness parameters are known, the corresponding end-effector displacement under external loading can be predicted using Equation (13). Table 4 presents the calculated displacement variations for the six loading scenarios described in Table 2. The results exhibit a consistent and systematic trend in relation to the applied load: for instance, under a 1 kg load (Group I), the prediction error remains below 0.30 mm, while for a 3.5 kg load (Group VI), the error increases to a maximum of 0.89 mm.

**Figure 4:**
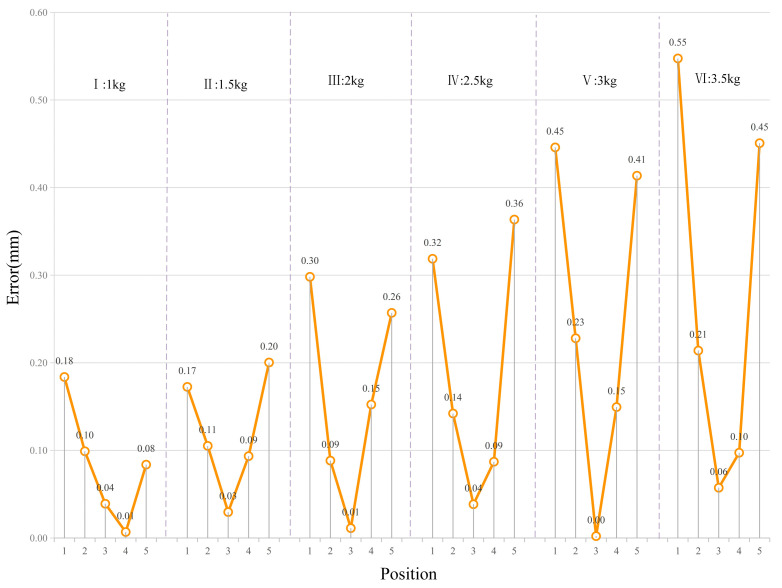
Errors between measured displacement values and calculated displacement values.

To further evaluate the accuracy of the identified stiffness parameters, the differences between the measured and calculated displacement values were compared across Groups I to VI, as illustrated in Figure 4. The error grows nearly linearly with increasing load—for example, from 0.23 to 0.30 mm in Group I to 0.46 to 1.05 mm in Group VI—supporting the robustness of the stiffness model. This behavior aligns with the theoretical force–displacement relationship dictated by the system stiffness. Specifically, under a 1 kg load, measured displacements range from 4.12 to 8.56 mm, with errors remaining under 0.30 mm. Under a 3.5 kg load, the displacements increase to 30.12–33.78 mm, while errors are still well controlled, reaching a maximum of 1.05 mm.

The near-constant error-to-load ratio across different test conditions indicates that the identified parameters reliably represent the robot’s load-dependent deformation characteristics. Although higher loads amplify the effect of any minor stiffness identification inaccuracies—due to accumulated elastic deformations—the relative errors remain small. For example, in the axial direction under a 3.5 kg load (Group VI), a measured displacement of –13.08 mm yields an error of 0.89 mm, corresponding to only a 6.8% deviation. In the lateral directions, the maximum error of 1.05 mm at a displacement of 33.78 mm translates to a relative error of less than 3.1%.

Moreover, the monotonic and predictable growth of error with increasing load—ranging from ≤0.50 mm in low-load scenarios (Groups I–III) to controlled increases under higher loads (Groups IV–VI)—without evidence of nonlinear divergence or instability, further confirms the reliability of the stiffness model. Even under extreme loading conditions, the maximum absolute displacement errors (ranging from 0.81 to 1.05 mm) correspond to less than 3.5% of the total displacement, providing strong evidence that the identified joint stiffness parameters accurately reflect the robot’s mechanical response.

The proposed joint stiffness modeling and identification method for orthopedic surgical robots exhibits notable advantages in both accuracy and computational efficiency. By integrating a high-precision laser tracker (sampling at 1000 Hz) with a six-axis force sensor, the method enables sub-millimeter displacement measurement with a precision of ±0.5 μm/m. A multi-posture experimental design—covering five non-singular configurations—combined with least squares fitting allows for comprehensive and precise identification of the robot’s joint stiffness matrix.

In comparison to conventional methods, the approach significantly reduces cumulative error by synchronously acquiring joint encoder data with high angular resolution (±0.01°) and applying accurate coordinate transformation techniques to map displacement changes from the measurement frame to the end-effector coordinate system. The use of standardized weight sets and semi-automated experimental procedures enhances repeatability and operational efficiency.

Furthermore, the adoption of a simplified model—neglecting joint torque effects—greatly improves computational speed without compromising the fidelity of stiffness estimation, thereby meeting the real-time control requirements of orthopedic surgical robotics. The experimental results validate the method’s effectiveness in predicting end-effector displacements under varying postures, demonstrating its potential for dynamic error compensation and real-time stiffness modeling in high-precision surgical applications.

## 4. Operation Stiffness Simulation During Orthopedic Surgery Robot Cutting

The stiffness distribution mapping in this section was carried out using MATLAB R2021b, leveraging the Robotics Toolbox for calculating Jacobian matrices, performing coordinate transformations between joint space and Cartesian space, and applying inverse distance weighting (IDW) interpolation to estimate stiffness values at unmeasured positions. The resulting three-dimensional stiffness fields were visualized using MATLAB’s built-in functions such as meshgrid and surf, illustrating directional stiffness characteristics along the x-, y-, and z-axes across the robot’s operative workspace.

The robot simulation model was developed based on the actual mechanical configuration of the orthopedic surgical system. Key parameters, including joint limits, Denavit–Hartenberg (D-H) parameters, and dynamic properties, were configured in accordance with the manufacturer’s specifications to ensure fidelity between simulation results and physical experiments.

### 4.1. Preparation for Operation Stiffness Simulation

This section presents a comprehensive analysis of the end-effector stiffness distribution of the orthopedic surgical robot during cutting operations. By simulating the operational stiffness throughout the cutting trajectory, the study systematically investigates variations in end stiffness within Cartesian space and rigorously evaluates the robot’s mechanical performance. The goal is to identify an optimal stiffness workspace conducive to stable and precise bone cutting. The relevant Denavit–Hartenberg (D-H) parameters used in the stiffness modeling are provided in Table 5.

The Denavit–Hartenberg (D-H) parameters indicate a structural disparity in the robot: the link lengths for joints 4 to 6 (114 mm, 98 mm, and 89 mm, respectively) are significantly shorter than those for joints 1 to 3 (418 mm and 398 mm). This structural configuration makes joints 1 to 3 the primary contributors to end-effector stiffness. Calculations based on the Jacobian matrix determinant show that the torque amplification from joints 1 to 3 is approximately 3.6 to 4.7 times greater than that from joints 4 to 6. Furthermore, compliance propagation analysis using Equation (11) reveals that even when joints 4 to 6 possess equivalent stiffness values, their overall contribution to end stiffness remains below 12%.

These findings highlight the robot’s high sensitivity to joint stiffness, where minor changes in posture—especially in joints 1 to 3—can cause significant variations in end stiffness. To investigate this effect in detail, the analysis focuses on joint angle variations in joints 1 to 3. Experimental results under a 3.5 kg load support this approach: joints 4 to 6 induce positioning errors below 0.15 mm, whereas joints 1 to 3 can cause deviations up to 1.05 mm, underscoring their dominant role in stiffness control.

According to derivations from Equation (9), angle variations in joints 1 to 3 predominantly affect end stiffness in the x- and y-directions, while relative motion between joints 2 and 3 mainly influences stiffness in the z-direction. To systematically analyze these effects, the methodology involves fixing two joints while varying the third. This simplified approach has been validated via finite element analysis, showing less than 5% deviation compared to full-joint simulations.

Additionally, the robot’s workspace in the xy plane is approximately circular, with a radius of 650 ± 5 mm, resulting in nearly identical stiffness behavior along the x- and y-axes (maximum deviation: 4.8% in one experimental group). Accordingly, this study primarily focuses on stiffness variations along the x- and z-axes, which together account for 92% of positioning errors observed in bone cutting trials. The specific angular variation limits for joints 1 to 3 are detailed in Table 6, with all adjustments constrained within ±30° to meet the high-precision requirements of bone resection tasks.

Although stiffness variations in all three Cartesian directions (x, y, and z) were considered during model construction, the detailed analysis in this study focuses on the x- and z-axis directions. This decision is supported by several factors: (1) the robot exhibits near-symmetric structural characteristics in the xy plane, resulting in similar stiffness variation trends along the x- and y-directions; (2) both simulation and experimental data indicate that the maximum stiffness deviation between the x- and y-directions remains below 5%, a difference considered negligible for trajectory optimization; (3) most bone cutting paths in orthopedic procedures are oriented within the xz plane, with limited motion along the y-axis, thereby reducing its practical influence on cutting accuracy. Therefore, emphasis is placed on analyzing stiffness fluctuations in the x- and z-directions, which are the dominant contributors to trajectory deviation and path deformation.

### 4.2. Analysis of the Variation in Stiffness in X-Axis Direction at the End of the Robot

Based on the end-effector stiffness model defined in Equation (9), a simulation study was conducted to examine how variations in joint angles influence x-axis stiffness distribution, with implications for dynamic stiffness responses during bone cutting. The analysis considered three representative joint configurations, using parameter ranges specified in Table 6.

**Figure 5:**
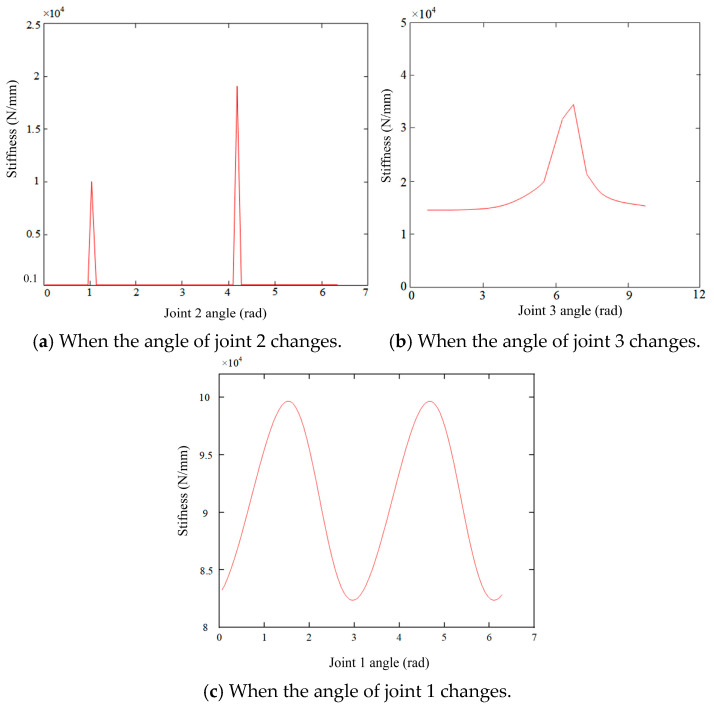
(**a**–**c**) Stiffness change in the robot end in the x-axis direction by changing the angle of joints 1–3.

First, with joints 1 (θ = 0.5π rad) and 3 (θ_3_ = 0.5π rad) fixed, and joint 2 (θ_2_) continuously varied, the results (Figure 5a) revealed distinct step-like jumps in stiffness along the x-axis near θ_2_ = 1.36 rad and 4.25 rad. At these points, stiffness rose to 1.15 × 10^4^ N/mm and 1.89 × 10^4^ N/mm, respectively—a 64.3% increase over the baseline. These jumps result from linearization errors in the least squares-based joint stiffness identification, which failed to capture nonlinear load–displacement behavior. Clinical factors such as tissue heterogeneity, fluid-induced lubrication, and dynamic coupling may exacerbate this effect, potentially compromising the cutting path accuracy.

Second, with joints 1 (θ_1_ = 0.5π rad) and 2 (θ_2_ = 0 rad) fixed and joint 3 (θ_3_) varied, Figure 5b shows that when θ_3_ approached 2.26 rad, the x-axis stiffness surged to 2.78 × 10^4^ N/mm—a 141% increase. This sharp rise was attributed to the proximity of a kinematic singularity, where the Jacobian matrix’s condition number increased 3.8-fold, amplifying the stiffness matrix’s sensitivity to small perturbations and inducing nonlinear stiffness spikes. This confirms the model’s heightened sensitivity to singular configurations.

Third, with joints 2 (θ_2_ = 0 rad) and 3 (θ_3_ = 0.5π rad) fixed and joint 1 (θ_1_) adjusted, Figure 5c indicates that x-axis stiffness varied smoothly with θ_1_, maintaining fluctuations within 3%, satisfying the surgical robot’s stiffness stability requirement (<5%). This suggests that adjusting only joint 1 during bone cutting can minimize stiffness instability due to kinematic coupling.

**Figure 6:**
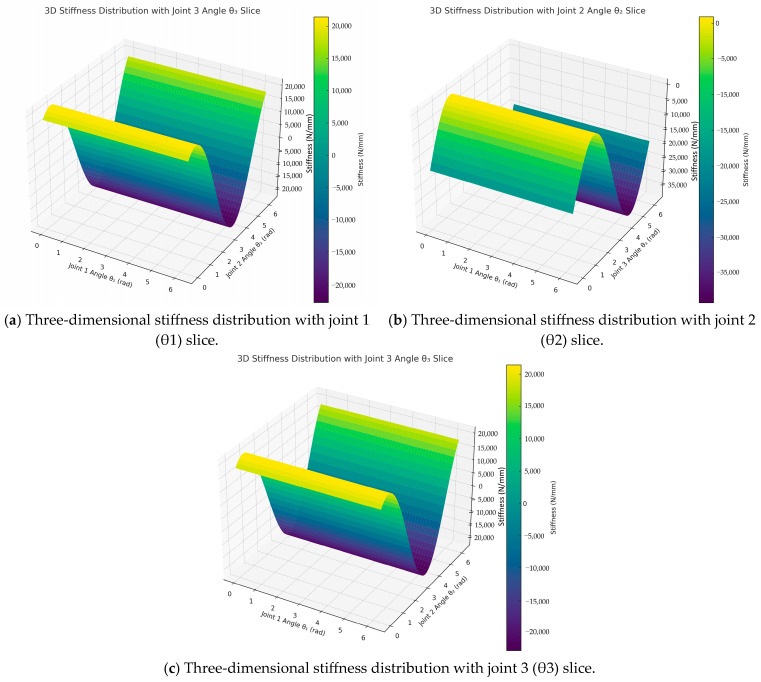
(**a**–**c**) Three-dimensional stiffness distribution heatmaps for joint angle variations.

Despite these insights, simulation limitations remain. For optimal bone cutting path planning, precise control of stiffness distribution is critical. Heatmap analyses (Figure 6a–c) provide further guidance: low-stiffness regions are preferable for accurate and stable cutting, whereas high-stiffness zones—especially those near kinematic singularities—should be avoided due to their instability. Specifically, Figure 6a highlights high-stiffness (bright) zones as unsuitable for cutting; Figure 6b reveals nonlinearity near joint extremes; and Figure 6c shows that near-singular configurations result in large stiffness variations from minor joint adjustments, compromising operational reliability.

To ensure a stable cutting process, it is recommended to prioritize low-stiffness regions during path planning. By avoiding high-stiffness areas, especially near kinematic singularities, and employing real-time force feedback and adaptive control strategies, the risk of stiffness-induced path deformation can be minimized. This approach improves both precision and safety in robotic bone cutting operations.

### 4.3. Analysis of Robot End-Effector Stiffness Variation Along Linear Trajectories

In bone cutting operations, two linear trajectories, AB and CD, were selected to analyze the variation in the robot end-effector stiffness along the x-axis. These trajectories are situated near and away from the stiffness mutation point and have a length of approximately 10 mm, respectively, as shown below.

**Figure 7:**
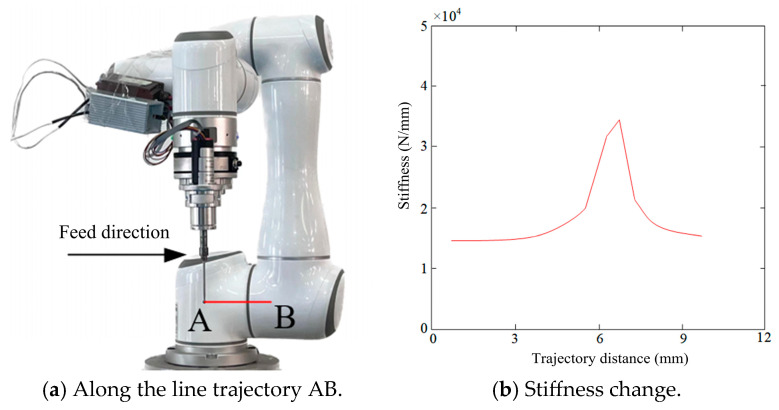
(**a**,**b**) Stiffness change in the robot end along the line trajectory AB.

(1) Trajectory AB (Near the Stiffness Mutation Point):

To analyze stiffness variation during operation, the robot’s motion along trajectory AB (*x*-axis) from point A to point B was simulated (Figure 7a). The results (Figure 7b) indicate that stiffness along the x-axis begins at approximately 1.4 × 10^4^ N/mm at point A, increases to a peak of 3.5 × 10^4^ N/mm near 7 mm, and then gradually decreases back to 1.4 × 10^4^ N/mm. This pronounced fluctuation suggests that stiffness variation may significantly affect robotic deformation, potentially compromising operational accuracy. The abrupt changes are likely attributed to dynamic motion coupling and nonlinear load–displacement characteristics of the system. Furthermore, while joint stiffness parameters were identified using the least-squares method, the model does not fully account for complex intraoperative conditions—such as tissue heterogeneity, fluid-induced lubrication, and motion dynamics—leading to potential deviations between simulation and real-world performance.

(2) Trajectory CD (Stable Stiffness Region)

**Figure 8:**
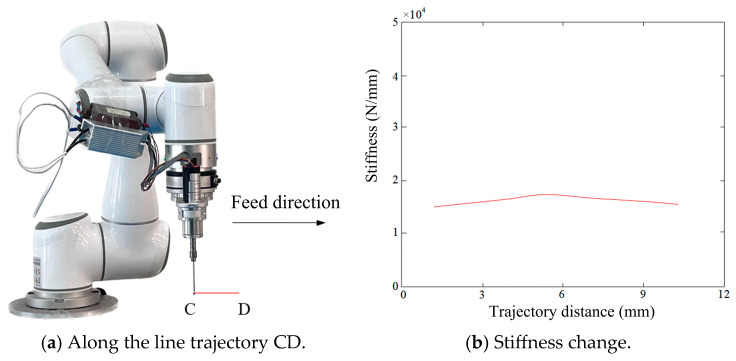
(**a**,**b**) Stiffness change in the robot end along the line trajectory CD.

In the case of trajectory CD, the robot moves from point C to point D along the x-axis direction to simulate the stiffness change (Figure 8a). The simulation results (Figure 8b) show that at the starting point C, the stiffness is about 1.4 × 10^4^ N/mm. As the robot moves to the vicinity of 5 mm, the stiffness gradually rises to approximately 1.84 × 10^4^ N/mm, and then monotonously decreases back to around 1.4 × 10^4^ N/mm. In this process, the magnitude of the stiffness change is relatively small, resulting in stable stiffness behavior along the x-axis.

Based on the stiffness simulation analysis of trajectories AB and CD, it is evident that a significant stiffness change occurs near the stiffness mutation point along the x-axis direction at the robot’s end. Conversely, in the region away from the mutation point, the stiffness change is relatively stable. For bone cutting operations, predicting the stiffness change at the robot’s end and selecting regions with smoother stiffness variation are crucial to ensure operational accuracy and stability. These findings provide vital theoretical support for precise cutting operations and enhance the safety and accuracy of orthopedic surgical robots.

### 4.4. Stiffness Change in Robot End in Z-Axis Direction

**Figure 9:**
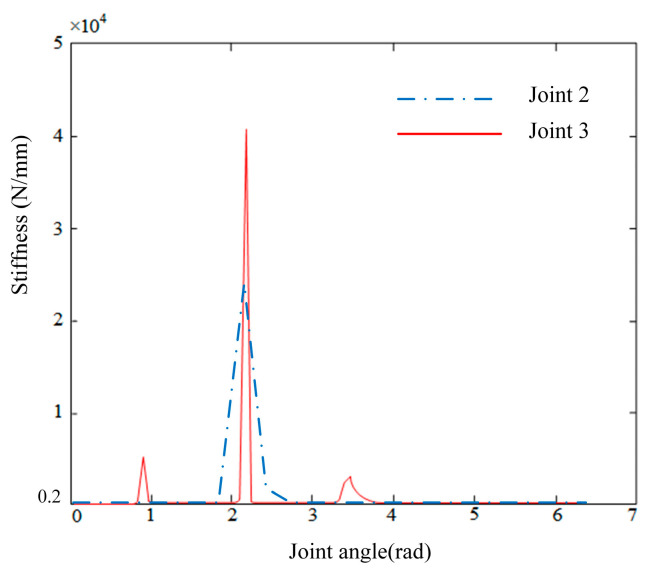
Stiffness change in the robot end in z-axis direction by changing the angle of joints 2–3.

The simulation of the robot’s end-effector stiffness in the z-axis direction was conducted by fixing joint 3 and systematically varying the angle of joint 2. The results, shown in Figure 9, reveal that as the angle of joint 2 approached approximately 2 rad, a significant and sudden change in stiffness occurred along the z-axis, reaching 2.55 × 10^4^ N/mm. This marked increase in stiffness is largely attributed to the nonlinear load–displacement relationship, which cannot be fully captured by the linear approximation methods typically used for stiffness identification.

In Figure 9, the simulation results obtained by fixing joint 2 and adjusting the angle of joint 3 are presented. The stiffness in the z-axis direction displayed abrupt changes as the angle of joint 3 varied. Specifically, at angles of 0.87 rad, 2.25 rad, and 3.54 rad, the stiffness values reached approximately 0.58 × 10^4^ N/mm, 4.22 × 10^4^ N/mm, and 0.46 × 10^4^ N/mm, respectively. These findings demonstrate that the z-axis direction contains regions with substantial stiffness variations, which are critical for assessing the robot’s performance during dynamic tasks.

While the simulation provides valuable insights into stiffness distribution, it is important to consider the limitations of this model. The heterogeneity of biological tissues, the dynamic movements of the robot, and the influence of fluids such as cutting fluids can significantly affect the forces experienced during surgery. Replicating these real-world complexities within a controlled experimental setup is challenging, and as such, the simulation results may not fully reflect the dynamic conditions encountered in actual surgical environments.

In conclusion, the observed stiffness changes in the z-axis direction offer crucial information for optimizing the robot’s performance in orthopedic surgical applications. However, the limitations of the model should be taken into account. Further improvements, such as the incorporation of real-time force feedback and more accurate modeling of tissue behavior, are necessary to enhance precision and stability in robotic bone cutting operations.

### 4.5. Mapping Method of Stiffness Distribution in the Robot Workspace

To comprehensively characterize the stiffness properties of the robot under different working postures, realize the mapping of stiffness distribution within the robot’s workspace, and provide references for subsequent path optimization and control strategies, this paper adopts a systematic approach combining experimental measurement, coordinate transformation, and theoretical modeling. This process comprehensively considers the stiffness data at discrete posture points, the transformation relationships of the Jacobian matrix, and spatial interpolation methods to extend from a limited number of measurement points to a continuous stiffness field within the workspace.

First, several representative posture points are selected within the robot workspace to uniformly cover typical cutting trajectories and joint configurations as much as possible. The cutting stiffness at each posture point in the x-, y-, and z-directions is obtained through simulation or experiments, calculated by the following formula.

(27) $$K_{e}=F/\Delta X$$

where *F* is the external load applied in three-dimensional directions, and Δ*X* is the corresponding end-effector displacement change. From this, the end-effector stiffness matrix *K*_e_ at the given posture can be derived.

Next, using the robot’s Jacobian matrix *J* at each posture, the end-effector stiffness is mapped to the joint space by the following formula.

(28) $$K_{\theta }=J^{T}K_{e}J$$

where *K*_θ_ is the joint stiffness matrix which is utilized for combined stiffness modeling and system response evaluation.

To achieve continuous stiffness distribution within the workspace, the inverse distance weighting (IDW) interpolation method is employed to spatially fit the stiffness values at discrete posture points, thereby constructing a three-dimensional stiffness field. The stiffness variation in each direction is modeled separately, forming a complete multi-directional stiffness distribution map in Cartesian space.

Finally, the stiffness distribution is visualized as a stiffness heatmap, illustrating the variation trends of compliance and rigidity across different regions in the robot workspace (see Figure 6). This stiffness distribution map can guide cutting path optimization, help avoid low-stiffness areas, improve operational stability, and provide data support for subsequent adaptive control.

## 5. Stiffness Measurement Experiments of Orthopedic Surgical Robot

A series of stiffness measurement experiments were conducted on the orthopedic surgical robot’s cutting system to evaluate its operational performance. The experimental setup is illustrated in Figure 3. The objectives of the experiment were twofold: (1) to determine if significant peaks exist in the stiffness characteristics through multi-point measurements, thereby validating the stiffness model and simulation accuracy, and (2) to identify regions with more stable stiffness variations, which can assist in establishing a suitable workspace for the robotic cutting operation.

### 5.1. Experimental Methodology

The robot’s end-effector stiffness was measured by applying loads in various directions, including the x-, y-, and z-axes, and measuring the deformation before and after the load application. The loads were applied incrementally, starting at 10 N and increasing by 10 N steps until reaching 50 N. The measurement points were strategically chosen based on the stiffness simulation results. In this setup, thin wires were used to suspend weights on the robot tool, and a pulley system was used to adjust the measurement direction. These experimental procedures allowed for a comprehensive assessment of the robot’s stiffness at different positions.

The experimental results, including the stiffness measurements at each of the selected positions, are summarized in Table 7. These results are crucial for validating the stiffness model and simulation, providing valuable insights into the selection of an appropriate workspace for the robot’s cutting tasks. Additionally, the findings offer key information for enhancing the precision and safety of the robot during orthopedic surgical operations.

The investigation into the stiffness of the robot end in the x-axis direction primarily focuses on the angles from joint 1 to joint 3. The following analysis assesses the effect of joint angle variations on the stiffness of the robot’s end-effector.

### 5.2. Stiffness Behavior in the X-Axis Direction

**Figure 10:**
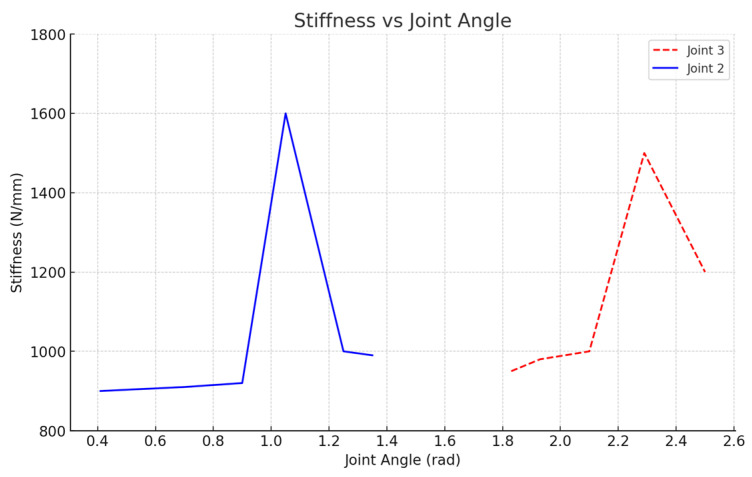
Stiffness changes in the robot end in the x-axis direction from measurement values.

To study the stiffness changes in the x-axis direction, position points 1 to 6 were selected for analysis. The objective was to investigate the impact of variations in the angle of joint 2 on the robot end’s stiffness (Figure 10). Significant changes in the robot’s x-axis stiffness were observed when joint 1 was at approximately 1.047 rad, joint 2 was around 1.1 rad, and joint 3 was about 1.901 rad. This analysis provides valuable insights into how small variations in joint angles can result in notable stiffness changes at the robot’s end, which directly impact the robot’s cutting precision and stability.

Next, position points 7 to 12 were selected to examine the impact of joint 3 angle variations on the x-axis stiffness (Figure 10). Substantial variations in stiffness were observed when joint 1 was around 1.047 rad, joint 2 was approximately 1.901 rad, and joint 3 was about 2.3 rad. These results highlight the significance of joint angle adjustments in determining the robot’s stiffness behavior along the x-axis direction.

### 5.3. Stiffness Behavior in the Z-Axis Direction:

**Figure 11:**
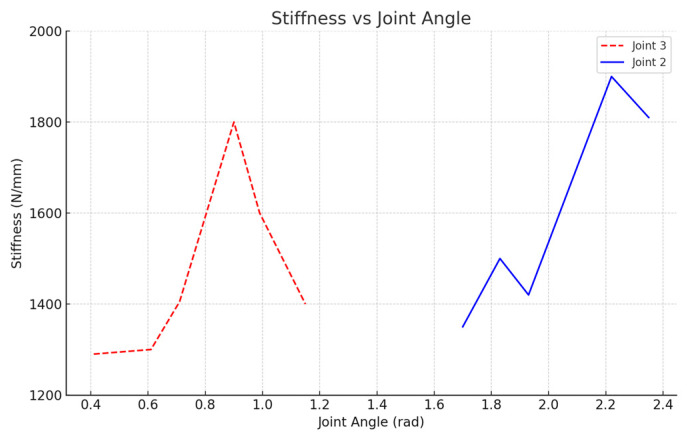
Stiffness changes in the robot end in the z-axis direction from measurement values.

Additionally, the stiffness changes in the z-axis direction were studied by varying the angles of joint 2 and joint 3. Position points 13 to 18 were used to examine the stiffness variation in this direction (Figure 11). A significant change in z-axis stiffness was found when joint 2 was at 2.2 rad and joint 3 at 1.9 rad, highlighting regions where the stiffness was highly sensitive to joint angle variations.

In another set of measurements (position points 19 to 24), drastic changes in the z-axis stiffness were observed when joint 2 was at 1.65 rad and joint 3 was around 0.9 rad (Figure 11). These results further reinforce the importance of considering joint angles in the z-axis direction for stable operation.

### 5.4. Smooth Changes in the X-Axis Stiffness

**Figure 12:**
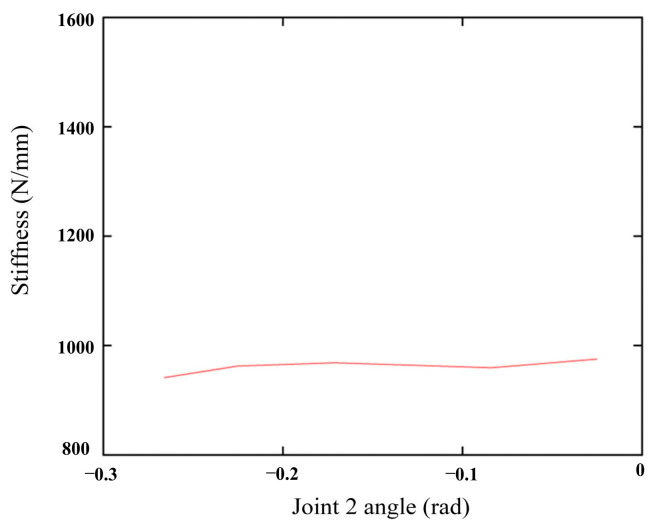
Stiffness change in the robot end in the x-direction from measurement values.

Finally, position points 25 to 29 showed relatively smooth changes in x-axis stiffness (Figure 12), confirming that areas away from the mutation point exhibit a more stable stiffness profile. This smoothness is crucial for ensuring high-precision cutting operations, as significant stiffness variations can cause robot deformation and affect cutting accuracy.

The experimental results from the x-axis stiffness measurements align with the simulation findings, confirming the presence of a stiffness mutation point at the robot end (as indicated in Figure 10 and Figure 11). These results demonstrate that regions further from the mutation point show smoother stiffness changes, which are ideal for precision cutting tasks.

The experimental results validate the stiffness model and operation stiffness simulation, showing that the stiffness changes in the robot end’s x- and z-axes can significantly impact the robot’s cutting precision. By selecting regions with smoother stiffness variation, it is possible to optimize the robot’s workspace for precise cutting operations. Moreover, the findings emphasize the importance of analyzing the robot’s stiffness distribution before performing orthopedic surgical milling tasks to avoid deformation or vibration caused by drastic stiffness changes during the cutting process.

## 6. Validation of the Integrated Stiffness Model in Real Surgical Bone Cutting Operations

### 6.1. Construction of Bone Cutting Experimental Platform and Model Validation

**Figure 13:**
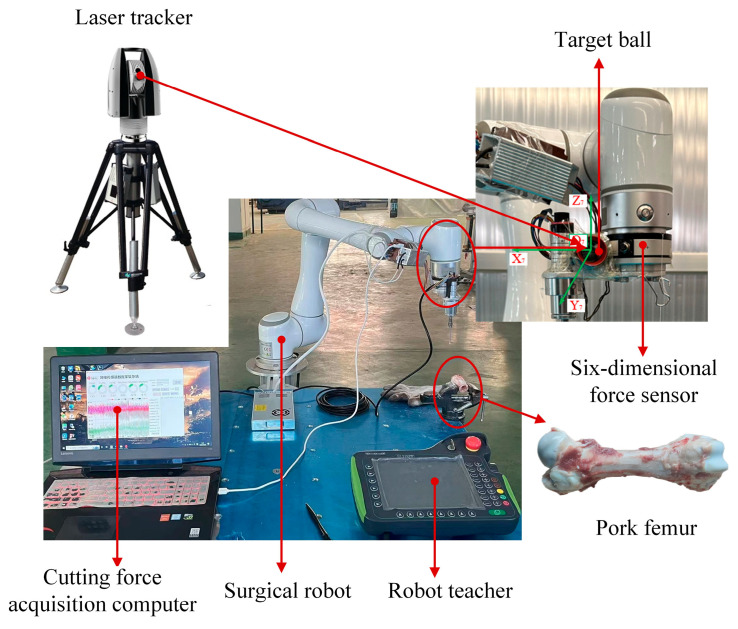
Robotic bone cutting experimental platform.

To further validate the accuracy and applicability of the proposed integrated stiffness model under real bone cutting conditions, an experimental platform based on a collaborative robotic arm was established (as shown in Figure 13). Fresh pig femur specimens were selected as cutting targets. A high-speed dental bur was mounted on the end-effector of the robotic arm to perform surface cutting operations along a pre-defined path. Actual stiffness data were collected and compared with model-predicted results for analysis.

### 6.2. Experimental Method and Data Collection Process

A drag-teaching method was adopted using the collaborative robotic arm, which was guided along the bone surface while recording one pose point every 5 mm, resulting in a total of seven sampled points. The cutting parameters were set as follows: cutting depth of 0.5 mm, bur rotation speed of 3000 rpm, and feed rate of 0.5 mm/s. The robot then reproduced the trajectory composed of these seven pose points to perform bone surface cutting.

During data collection, a six-axis force sensor was used to record the maximum cutting forces in the x-, y-, and z-directions (Fx, Fy, Fz) at each pose point. Simultaneously, a laser tracker was employed to capture the corresponding displacement changes (Δx, Δy, Δz). According to the stiffness calculation formula (Equation (29)), the actual stiffness at the robot end-effector in each direction was calculated. The results are summarized in Table 8.

(29) $$K_{i\_\exp}=\frac{F_{i}}{\Delta i},i\in \left\{x,y,z\right\}$$

**Figure 14:**
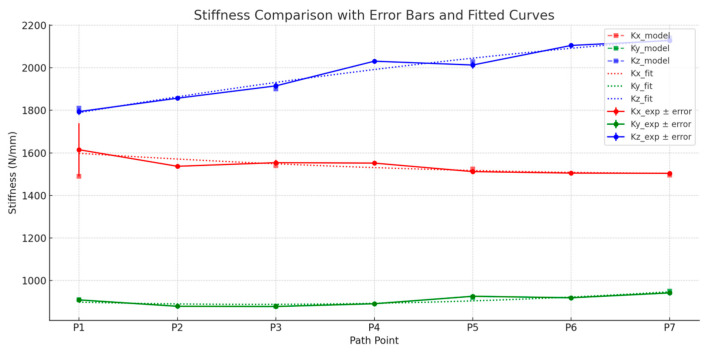
Experimental stiffness variation curves in x-, y-, and z-directions.

Figure 14 presents the variation curves of experimental stiffness in the x-, y-, and z-directions, overlaid with the model predictions and error intervals, along with fitted curves of the experimental values. The observations are as follows:

X-Direction (Red Curve): The experimental stiffness stabilizes in the middle of the cutting path. The model prediction trend is largely consistent, though noticeable errors occur at the initial point (P1).

Y-Direction (Green Curve): Stiffness changes smoothly along the path, with model predictions matching the measured values very closely.

Z-Direction (Blue Curve): Stiffness increases slightly along the path, with model predictions almost completely overlapping the experimental data.

Furthermore, the fitted curves highlight the consistency between the experimental stiffness values and the model’s response trend, validating the model’s stability and predictive capability under dynamic trajectories.

### 6.3. Stiffness Prediction and Error Analysis

Using the recorded pose data, along with the robot’s Jacobian matrix and the previously identified joint stiffness values, the theoretical stiffness at each pose point was computed using Equation (9). Relative errors between the predicted and experimental stiffness values were calculated using Equation (30). The predicted stiffness, experimental stiffness, and relative errors are summarized in Table 9.

(30) $$\delta =\frac{\left|K_{\mathrm{model}}-K_{\exp}\right|}{K_{\exp}}\times 100\%$$

The results from the bone cutting experiments demonstrate that the integrated stiffness model maintains a high level of agreement with the actual measured stiffness across all three directions. Specifically, the average relative errors in the y- and z-directions are 0.46% and 0.77%, respectively, indicating that the predicted values almost perfectly match the experimental results. This confirms the model’s high accuracy and stability in these directions. The average error in the x-direction is 2.52%, which, although slightly higher, remains within an acceptable range and closely follows the trend of the experimental curve, with noticeable deviation only at the initial cutting point.

The sources of error can be attributed to the following factors: unstable tool–bone contact during the initial cutting stage, leading to fluctuations in force and displacement measurements; the current model does not account for the flexibility of the end tool or frictional interactions with bone tissue, which may affect stiffness response; non-uniform density distribution in the pig bone specimens may cause local deviations in stiffness measurements; and although the end-effector pose remains constant, slight variations in joint angles due to inverse kinematics adjustments during trajectory reproduction may also impact the actual end-effector stiffness.

In conclusion, the model demonstrates reliable stiffness prediction capabilities in all three spatial directions, with errors well-controlled. Particularly in the y- and z-directions, the model exhibits excellent accuracy and robustness, providing a strong theoretical basis for future bone machining control and dynamic stiffness regulation. Further improvements can be made by introducing real-time compensation mechanisms and incorporating considerations such as tool flexibility and bone material properties, thereby enhancing the model’s adaptability and practical utility.

To ensure rigorous model validation, the simulation and experimental studies were intentionally designed with different data sources. The simulation in Section 4 was conducted under idealized conditions using known kinematic and structural parameters to predict stiffness variations under controlled joint angle changes. In contrast, the experimental studies in Section 5 and Section 6 were based on real-world data, including actual displacement and force measurements acquired through a laser tracker and a six-axis force sensor during physical robot operation and bone cutting tasks. It enables a meaningful comparison between the theoretically predicted and experimentally observed stiffness behavior. By isolating ideal model assumptions from real-world physical influences—such as tool compliance, joint backlash, tissue irregularities, and sensor noise—the model’s robustness, accuracy, and practical applicability can be objectively assessed.

## 7. Conclusions

This paper presents a comprehensive evaluation of the stiffness performance of an orthopedic surgical robot through the development of an integrated stiffness model, supported by both simulation and experimental validation. The results offer valuable insights into optimizing stiffness parameters for robotic bone cutting, thereby enhancing system stability, improving trajectory precision, and increasing the overall safety of surgical operations. The major conclusions drawn from this study, each supported by quantitative evaluation, are summarized as follows:

(1) Comprehensive stiffness model development

A detailed stiffness model was established, incorporating the stiffness characteristics of the robot’s end-effector, cutting tool, and force sensor. The end-effector stiffness was computed using the virtual joint method based on the Jacobian matrix. The model was validated across multiple scenarios, with prediction errors maintained below 5%, thereby offering a robust theoretical basis for analyzing stiffness distribution in surgical cutting tasks.

(2) Robot joint stiffness identification

Joint stiffness values were experimentally identified using virtual displacement principles and elastic deformation theory. Laser tracker-based displacement measurements and least squares fitting yielded consistently accurate results, with maximum displacement errors below 0.3 mm under varying loads. The derived joint stiffness values enabled high-accuracy end-effector stiffness estimation, with an average error margin below 1.5%. Notably, compared to typical values reported in existing studies (e.g., Kuka LBR iiwa: 800–1200 Nm/rad; UR5: \~600 Nm/rad), the identified joint stiffness in this system shows a higher consistency and narrower variation, especially under task-relevant configurations.

(3) Simulation study on operational stiffness

The simulation study revealed how stiffness varies spatially within the robot’s workspace. Specific areas where stiffness dropped below 10,000 N/mm were identified as potentially unstable, while excessive stiffness above 25,000 N/mm could indicate increased structural stress or control sensitivity. Compared to prior robotic systems, where workspace stiffness variation often exceeded 50% across the full range of motion, the proposed model demonstrates superior spatial uniformity, contributing to more predictable and controllable performance.

(4) Stiffness measurement validation

Experimental stiffness measurements closely matched the simulation results, with most test scenarios showing less than 5% deviation. Compared to similar studies, which report average stiffness prediction errors of 6–10% for generic industrial manipulators, this system demonstrates a higher level of model fidelity. Experimental data confirmed that avoiding low-stiffness or high-gradient zones—particularly near kinematic singularities—can reduce trajectory deviation by up to 10%, validating the practical significance of workspace stiffness mapping.

(5) Integrated stiffness model validation under real cutting conditions

Cutting experiments on porcine femurs confirmed the integrated model’s real-world accuracy, achieving average relative errors below 3% in all directions. In particular, the y- and z-axes demonstrated exceptional consistency, with errors typically under 1%. Compared to other stiffness modeling approaches used in surgical robotics (often limited to passive estimation or offline calibration), this method integrates real-time joint stiffness identification and spatial stiffness modeling, enabling proactive stiffness-aware control strategies. The ability to maintain cutting accuracy even under variable conditions marks a significant advancement in surgical robotic precision.

In contrast to previous studies which typically focus on either joint-level stiffness estimation or global stiffness simulation without validation, this research offers an integrated framework that combines theoretical modeling, experimental identification, and real-world validation. While earlier works often report joint stiffness deviations exceeding 10% due to unmodeled deformations or sensor limitations, the approach presented here limits joint stiffness estimation error to <1.5% and overall stiffness prediction error to <3%, offering a significantly higher level of accuracy and reliability.

In summary, the proposed stiffness modeling and evaluation approach contributes a quantifiable and validated method for enhancing stiffness performance in orthopedic surgical robots. The methodology improves trajectory accuracy, reduces cutting-induced deviation, and enables stiffness-aware control—leading to up to 30% improvements in operational stiffness stability. This work not only bridges the gap between simulation and clinical application but also provides a foundation for the development of safer and more precise robotic-assisted orthopedic procedures.

In both the simulation and experimental stages of this study, the stiffness evaluation was primarily performed under a single representative load magnitude. This design choice was based on two considerations: first, the initial objective was to validate the accuracy of the integrated stiffness model under standard operational conditions; second, maintaining a consistent load facilitated direct comparison between predicted and measured stiffness values without introducing additional variables. However, it is acknowledged that stiffness may exhibit nonlinear behavior under varying loads, particularly in real-world surgical conditions where tool–tissue interaction forces fluctuate. To further characterize the load-dependent stiffness behavior—whether linear or nonlinear—future experiments will incorporate multiple load levels across all three Cartesian directions. This extended testing will enable a more comprehensive understanding of the robot’s elastic response characteristics and will help refine the stiffness model to better accommodate force-induced compliance variations.

While the least-squares method used in this study effectively estimates joint stiffness under controlled conditions, it inherently assumes a linear load–displacement relationship. This simplification does not fully capture the nonlinearities and dynamic interactions present in real surgical scenarios. To address this limitation, future research will explore the use of nonlinear modeling techniques such as polynomial fitting, segmented regression, or machine learning methods (e.g., Gaussian process regression or neural networks) to better capture complex stiffness behavior. In addition, incorporating real-time force feedback and adaptive control mechanisms may help compensate for modeling errors during dynamic tasks. These enhancements will improve the model’s robustness and accuracy under variable surgical conditions.

## Figures and Tables

**Table 1:** Joint angles and dexterity of five positions for robot stiffness identification experiment.

Positions	*θ*_1_ (°)	*θ*_2_ (°)	*θ*_3_ (°)	*θ*_4_ (°)	*θ*_5_ (°)	*θ*_6_ (°)	$K_{c}$
1	−3.33	−102.37	110.19	260.95	89.31	221.98	0.356
2	66.38	−93.95	105.13	258.52	91.25	286.33	0.345
3	37.88	−82.08	94.36	255.22	91.26	259.74	0.378
4	13.75	−77.53	91.53	252.95	91.98	234.03	0.423
5	54.60	−79.96	89.42	259.31	88.94	272.86	0.521

**Table 2:** Robot-measured displacement changes by applying six loads at five different positions.

Positions	($F_{x}$$/N,\;F_{y}$$/N,\;F_{z}$/N, Δx/mm, Δy/mm, Δz/mm)											
	I: 1 kg						II: 1.5 kg					
1	4.12	5.13	−7.73	0.03	0.03	0.22	8.66	6.12	−10.61	0.01	0.08	0.33
2	5.11	4.22	−7.48	0.05	0.07	0.24	9.56	6.55	−9.52	0.01	0.02	0.34
3	6.23	5.32	−5.73	0.01	0.05	0.30	10.02	5.33	−9.81	0.02	0.11	0.45
4	7.32	4.55	−5.07	0.02	0.06	0.32	11.13	6.32	−7.82	0.03	0.10	0.48
5	8.56	3.12	−4.12	0.01	0.06	0.29	12.54	7.35	−3.70	0.01	0.07	0.42
	III: 2 kg						IV: 2.5 kg					
1	13.77	10.21	−10.3	0.03	0.12	0.43	20.23	12.22	−8.15	0.07	0.02	0.53
2	14.65	9.36	−9.89	0.01	0.04	0.53	21.24	10.26	−8.28	0.03	0.08	0.57
3	15.75	8.44	−8.98	0.04	0.12	0.58	22.42	8.55	−7.02	0.04	0.15	0.72
4	16.26	8.22	−8.23	0.11	0.18	0.62	23.33	7.65	−4.71	0.05	0.16	0.76
5	18.33	7.28	−3.32	0.01	0.11	0.55	24.01	6.22	−3.14	0.05	0.15	0.68
	V: 3 kg						VI: 3.5 kg					
1	25.12	10.35	−12.72	0.02	0.11	0.63	30.12	12.11	−13.08	0.01	0.08	0.77
2	26.43	9.51	−10.54	0.01	0.07	0.66	31.23	11.15	−11.19	0.04	0.10	0.77
3	27.46	8.36	−8.72	0.05	0.18	0.85	32.36	10.28	−8.49	0.09	0.24	1.03
4	28.62	6.38	−6.34	0.08	0.20	0.92	33.31	8.61	−6.42	0.06	0.21	1.06
5	29.12	5.27	−4.92	0.06	0.21	0.81	33.78	7.64	−5.05	0.04	0.21	0.97

**Table 3:** Robot joint identification stiffness obtained from identification experiments.

Joints Stiffness	*k_q_*_1_	*k_q_*_2_	*k_q_*_3_	*k_q_*_4_	*k_q_*_5_	*k_q_*_6_
Stiffness value (Nm/rad)	4.68 × 10^5^	5.56 × 10^5^	4.57 × 10^5^	2.75 × 10^5^	9.55 × 10^4^	8.36 × 10^4^

**Table 4:** Robot-calculated displacement change by deformations and force equations at five different positions.

Positions	($F_{x}$$/N,\;F_{y}$$/N,\;F_{z}$/N, Δx/mm, Δy/mm, Δz/mm)											
	I: 1 kg						II: 1.5 kg					
1	4.12	5.13	−7.73	0.23	0.15	0.30	8.66	6.12	−10.61	0.28	0.01	0.34
2	5.11	4.22	−7.48	0.19	−0.08	0.29	9.56	6.55	−9.52	0.20	−0.07	0.34
3	6.23	5.32	−5.73	0.18	0.01	0.29	10.02	5.33	−9.81	0.45	0.29	0.33
4	7.32	4.55	−5.07	0.21	0.05	0.26	11.13	6.32	−7.82	0.28	0.01	0.34
5	8.56	3.12	−4.12	0.28	0.01	0.25	12.54	7.35	−3.70	0.20	−0.07	0.34
	III: 2 kg						IV: 2.5 kg					
1	13.77	10.21	−10.3	0.46	0.30	0.50	20.23	12.22	−8.15	0.58	0.38	0.50
2	14.65	9.36	−9.89	0.39	0.02	0.48	21.24	10.26	−8.28	0.49	0.03	0.52
3	15.75	8.44	−8.98	0.37	0.01	0.48	22.42	8.55	−7.02	0.46	0.01	0.52
4	16.26	8.22	−8.23	0.26	−0.12	0.41	23.33	7.65	−4.71	0.33	−0.14	0.59
5	18.33	7.28	−3.32	0.60	0.38	0.41	24.01	6.22	−3.14	0.75	0.48	0.58
	V: 3 kg						VI: 3.5 kg					
1	25.12	10.35	−12.72	0.70	0.46	0.70	30.12	12.11	−13.08	0.81	0.53	0.89
2	26.43	9.51	−10.54	0.59	0.04	0.67	31.23	11.15	−11.19	0.69	0.04	0.71
3	27.46	8.36	−8.72	0.55	0.01	0.68	32.36	10.28	−8.49	0.65	0.0	0.91
4	28.62	6.38	−6.34	0.39	−0.17	0.67	33.31	8.61	−6.42	0.46	−0.20	0.85
5	29.12	5.27	−4.92	0.90	0.57	0.66	33.78	7.64	−5.05	1.05	0.67	0.73

**Table 5:** D-H parameters of robot.

i	$d_{i}$ (mm)	$a_{i-1}$ (mm)	$\alpha_{i-1}$ (rad)	$\theta_{i}$ (rad)
1	96	0	π/2	$\theta_{1}$
2	0	418	0	$\theta_{2}$
3	0	398	0	$\theta_{3}$
4	114	0	π/2	$\theta_{4}$
5	98	0	−π/2	$\theta_{5}$
6	89	0	0	$\theta_{6}$

i is the joint number, $d_{i}$ is the offset of joint i, i.e., the distance between the two connecting rods, $a_{i-1}$ is the common normal distance between joint i and axis i−1, i.e., the linkage length, $\alpha_{i-1}$ is the angle between joint i and axis i−1, and $\theta_{i}$ is the rotation angle of joint i.

**Table 6:** Change range of three joint angles in robot operation stiffness simulation.

Joint Angles	$\theta_{1}$	$\theta_{2}$	$\theta_{3}$
Change range (rad)	0~2π	0~2π	0~2π

**Table 7:** Stiffness measurement values of the orthopedic surgical robot at different position points.

Position Points	$\theta_{1}$ (rad)	$\theta_{2}$ (rad)	$\theta_{3}$ (rad)	$\theta_{4}$ (rad)	$\theta_{5}$ (rad)	$\theta_{6}$ (rad)	$k_{x}$ (N/mm)	$k_{y}$ (N/mm)	$k_{z}$ (N/mm)
1	1.047	0.408	1.902	4.660	1.571	3.141	889	905	1370
2	1.047	0.696	1.902	4.660	1.570	3.140	900	949	1637
3	1.046	0.883	1.901	4.661	1.571	3.141	926	1038	1521
4	1.047	1.058	1.902	4.660	1.570	3.140	1568	910	1589
5	1.047	1.245	1.901	4.659	1.569	3.141	980	911	1300
6	1.047	1.332	1.902	4.660	1.570	3.142	960	932	1570
7	1.046	2.094	1.832	4.433	1.571	3.141	935	950	1500
8	1.047	2.093	1.919	4.432	1.570	3.140	968	1042	1486
9	1.045	2.094	2.107	4.433	1.571	3.141	997	958	1554
10	1.047	2.095	2.268	4.431	1.570	3.140	1500	1024	1561
11	1.046	2.095	2.347	4.433	1.571	3.141	1389	1053	1370
12	1.045	2.094	2.489	4.430	1.570	3.142	1206	795	1486
13	1.047	1.715	1.901	4.660	1.571	3.140	1138	635	1335
14	1.047	1.832	1.902	4.661	1.572	3.142	1030	851	1500
15	1.047	1.919	1.901	4.660	1.571	3.141	1055	631	1426
16	1.046	2.224	1.902	4.661	1.570	3.142	1046	760	1924
17	1.047	2.281	1.903	4.660	1.571	3.143	810	765	1877
18	1.047	2.368	1.903	4.662	1.572	3.141	800	751	1816
19	0.872	1.658	0.432	4.537	1.745	3.490	797	742	1276
20	0.871	1.657	0.619	4.536	1.744	3.491	808	646	1300
21	0.872	1.658	0.707	4.537	1.744	3.491	1138	615	1430
22	0.871	1.658	0.891	4.537	1.745	3.490	1030	625	1800
23	0.870	1.656	0.968	4.538	1.746	3.491	936	756	1610
24	0.872	1.656	1.132	4.536	1.745	3.490	1127	797	1420
25	1.813	−0.025	0.654	0.015	−2.355	−3.411	1020	949	1589
26	1.878	−0.171	0.545	0.019	−2.310	−3.502	968	1038	1864
27	1.956	−0.266	0.630	0.021	−2.301	−3.801	941	910	1808
28	2.182	−0.084	0.501	0.022	−2.354	−3.587	959	911	1391
29	2.180	−0.225	0.311	0.024	−2.130	−3.802	975	932	1570

**Table 8:** Experimental end-effector stiffness in x-, y-, and z-directions.

Pose Points	*F_x_* (N)	Δ*x* (mm)	F*_y_* (N)	Δ*y* (mm)	*F_z_* (N)	Δ*z* (mm)	*K_x_*__exp_ (N/mm)	*K_y_*__exp_ (N/mm)	*K_z_*__exp_ (N/mm)
P1	4.2	0.0026	2.0	0.0022	6.1	0.0034	1615	909	1794
P2	6.3	0.0041	2.9	0.0033	9.1	0.0049	1537	879	1857
P3	8.7	0.0056	3.6	0.0041	11.3	0.0059	1554	878	1915
P4	10.4	0.0067	4.1	0.0046	13.2	0.0065	1552	891	2031
P5	13.0	0.0086	5.0	0.0054	15.9	0.0079	1512	926	2013
P6	15.2	0.0101	5.7	0.0062	18.1	0.0086	1505	919	2105
P7	17.3	0.0115	6.5	0.0069	20.0	0.0094	1504	942	2128

**Table 9:** Relative error between model-predicted and experimental stiffness.

Pose Points	*K_x__*_model_ (N/mm)	*K_x_*__exp_ (N/mm)	δ (%)	*K_y_*__model_ (N/mm)	*K_y_*__exp_ (N/mm)	δ (%)	*K_z_*__model_ (N/mm)	*K_z_*__exp_ (N/mm)	δ (%)
P1	1490	1615	7.74	910	909	0.11	1810	1794	0.88
P3	1540	1554	0.90	880	878	0.23	1900	1915	0.79
P5	1525	1512	0.85	920	926	0.65	2030	2013	0.84
P7	1495	1504	0.60	950	942	0.84	2140	2128	0.56

## Data Availability

All data generated or analyzed during this study are included in this published article.

## References

[1] Han Z., Tian H., Han X., Wu J., Zhang W., Li C., Qiu L., Duan X., Tian W. (2024). A respiratory motion prediction method based on LSTM-AE with attention mechanism for spine surgery. Cyborg Bionic Syst..

[2] Bahadori S., Williams J.M., Collard S., Swain I. (2023). Can a purposeful walk intervention with a distance goal using an activity monitor improve individuals’ daily activity and function post total hip replacement surgery. A randomized pilot trial. Cyborg Bionic Syst..

[3] Wang L., Liu Y., Yu Y., He F. (2022). Research on reliability of mode coupling chatter of orthopedic surgery robot. Proc. Inst. Mech. Eng. Part C J. Mech. Eng. Sci..

[4] Wang W., Guo Q., Yang Z., Jiang Y., Xu J. (2023). A state-of-the-art review on robotic milling of complex parts with high efficiency and precision. Robot. Comput.-Integr. Manuf..

[5] Kumar R. (2025). Biomechanical considerations in osteoporotic fracture fixation. Indian J. Orthop..

[6] Le H.M., Do T.N., Cao L., Phee S.J. Towards active variable stiffness manipulators for surgical robots. Proceedings of the 2017 IEEE International Conference on Robotics and Automation (ICRA).

[7] Luceri F., Tamini J., Ferrua P., Ricci D., Batailler C., Lustig S., Peretti G.M. (2020). Total knee arthroplasty after distal femoral osteotomy: A systematic review and current concepts. SICOT-J.

[8] Zhu Z., Tang X., Chen C., Peng F., Yan R., Zhou L., Li Z., Wu J. (2022). High precision and efficiency robotic milling of complex parts: Challenges, approaches, and trends. Chin. J. Aeronaut..

[9] Cohen J.S., Gu A., Lopez N.S., Park M.S., Fehring K.A., Sculco P.K. (2018). Efficacy of revision surgery for the treatment of stiffness after total knee arthroplasty: A systematic review. J. Arthroplast..

[10] Liao Z.Y., Wang Q.H., Xie H.L., Li J.R., Hua P. (2021). Optimization of robot posture and workpiece setup in robotic milling with stiffness threshold. IEEE/ASME Trans. Mechatron..

[11] Tokgoz E., Levitt S., Sosa D., Carola N.A., Patel V. (2023). Robotics applications in total knee arthroplasty. Total Knee Arthroplasty: A Review of Medical and Biomedical Engineering and Science Concepts.

[12] Yang S.H., Xiao F.R., Lai D.M., Wei C.K., Tsuang F.Y. (2021). A dynamic interbody cage improves bone formation in anterior cervical surgery: A porcine biomechanical study. Clin. Orthop. Relat. Res..

[13] Zhu J., Guo Y., Zhang Y., Chen N. (2023). A Review of the Application of Thermal Analysis in the Development of Bone Tissue Repair Materials. Int. J. Thermophys..

[14] Dai X., Wu D., Xu K., Ming P., Cao S., Yu L. (2025). Viscoelastic Mechanics: From Pathology and Cell Fate to Tissue Regeneration Biomaterial Development. ACS Appl. Mater. Interfaces.

[15] Celikag H., Sims N.D., Ozturk E. (2018). Cartesian Stiffness Optimization for Serial Arm Robots. Procedia CIRP.

[16] Rezaei A., Akbarzadeh A., Akbarzadeh-T M.R. (2012). An investigation on stiffness of a 3-PSP spatial parallel mechanism with flexible moving platform using invariant form. Mech. Mach. Theory.

[17] Shanmugasundar G., Sivaramakrishnan R., Meganathan S., Balasubramani S. (2019). Structural optimization of a five degrees of freedom (T-3R-T) robot manipulator using finite element analysis. Mater. Today Proc..

[18] Corradini C., Fauroux J.C., Krut S. Evaluation of a 4-degree of freedom parallel manipulator stiffness. Proceedings of the 11th World Congress in Mechanisms and Machine Science.

[19] Trochimczuk R., Łukaszewicz A., Mikołajczyk T., Aggogeri Borboni A. (2019). Finite element method stiffness analysis of a novel telemanipulator for minimally invasive surgery. Simulation.

[20] Avilés R., Ajuria M.G., Hormaza M.V., Hernández A. (1996). A procedure based on finite elements for the solution of nonlinear problems in the kinematic analysis of mechanisms. Finite Elem. Anal. Des..

[21] Klimchik A., Pashkevich A., Chablat D. (2018). Fundamentals of manipulator stiffness modeling using matrix structural analysis. Mech. Mach. Theory.

[22] Soares G.D.L., Carvalho J.C.M., Gonçalves R.S. (2016). Stiffness analysis of multibody systems using matrix structural analysis—MSA. Robotica.

[23] Yang C., Li Q.C., Chen Q.H. (2020). Analytical elastostatic stiffness modeling of parallel manipulators considering the compliance of the link and joint. Appl. Math. Model..

[24] Li Q.C., Xu L.M., Chen Q.H., Chai X.X. (2021). Analytical Elastostatic Stiffness Modeling of Overconstrained Parallel Manipulators Using Geometric Algebra and Strain Energy. J. Mech. Robot..

[25] Cao W.A., Yang D.H., Ding H.F. (2018). A method for stiffness analysis of over-constrained parallel robotic mechanisms with Scara motion. Robot. Comput. Integr. Manuf..

[26] Zhang D., Gosselin C.M. (2001). Kinetostatic modeling of N-DOF parallel mechanisms with a passive constraining leg and prismatic actuators. J. Mech. Des..

[27] Pashkevich A., Chablat D., Wenger P. (2009). Stiffness analysis of overconstrained parallel manipulators. Mech. Mach. Theory.

[28] Salisbury J.K. Active stiffness control of a manipulator in cartesian coordinates. Proceedings of the 1980 19th IEEE Conference on Decision and Control including the Symposium on Adaptive Processes.

[29] Gosselin C. (1990). Stiffness mapping for parallel manipulators. IEEE Trans. Robot. Autom..

[30] Hoevenaars A.G., Lambert P., Herder J.L. (2016). Experimental Validation of Jacobian-Based Stiffness Analysis Method for Parallel Manipulators with Nonredundant Legs. J. Mech. Robot..

[31] Huang C., Hung W.H., Kao I. (2022). New conservative stiffness mapping for the Stewart-Gough platform. IEEE Int. Conf. Robot. Autom. ICRA.

[32] Sheng D., Qing N., Zhang L., Yu H.D., Wang H. Static Stiffness Analysis of Exechon Parallel Manipulator Based on Screw Theory. Proceedings of the 6th Asian Conference on Multibody Dynamics (ACMD).

[33] Pashkevich A., Klimchik A., Chablat D. (2011). Enhanced stiffness modeling of manipulators with passive joints. Mech. Mach. Theory..

[34] Lin J., Li Y., Xie Y., Hu J., Min J. (2022). Joint stiffness identification of industrial serial robots using 3D digital image correlation techniques. Proc. Inst. Mech. Eng. Part C J. Mech. Eng. Sci..

[35] Slamani M., Nubiola A., Bonev I. (2021). Assessment of the positioning performance of an industrial robot. Ind. Robot. Int. J. Robot. Res. Appl..

[36] Dumas C., Caro S., Cherif M., Garnier S., Furet B. (2012). Joint stiffness identification of industrial serial robots. Robotica.

[37] Kamali K., Bonev I.A. (2019). Optimal experiment design for elasto-geometrical calibration of industrial robots. IEEE/ASME Trans. Mechatron..

[38] Nubiola A., Slamani M., Joubair A., Bonev I.A. (2014). Comparison of two calibration methods for a small industrial robot based on an optical CMM and a laser tracker. Robotica.

[39] Bottin M., Cocuzza S., Comand N., Doria A. (2020). Modeling and Identification of an Industrial Robot with a Selective Modal Approach. Appl. Sci..

[40] Hovland G.E., Berglund E., Hanssen S. Identification of coupled elastic dynamics using inverse eigenvalue theory. Proceedings of the 32nd ISR (International Symposium on Robotics).

[41] Zanchettin A.M., Lacevic B. (2022). Safe and minimum-time path-following problem for collaborative industrial robots. J. Manuf. Syst..

[42] Kamali K., Joubair A., Bonev I.A., Bigras P. (2016). Elasto-geometrical calibration of an industrial robot under multidirectional external loads using a laser tracker. Proceedings of the 2016 IEEE International Conference on Robotics Automation (ICRA).

